# Characterization of gut microbiomes of household pets in the United States using a direct-to-consumer approach

**DOI:** 10.1371/journal.pone.0227289

**Published:** 2020-02-20

**Authors:** Aashish R. Jha, Justin Shmalberg, Jirayu Tanprasertsuk, LeeAnn Perry, Dan Massey, Ryan W. Honaker

**Affiliations:** 1 Research & Development Division, NomNomNow, Inc., Oakland, California, United State of America; 2 Department of Comparative, Diagnostic & Population Medicine, University of Florida, Gainesville, Florida, United States of America; University of Lincoln, UNITED KINGDOM

## Abstract

The role of gut microbiomes as important regulators of mammalian health is increasingly recognized, although feline and canine gut microbiomes remain poorly characterized. In this proof-of-concept study, we assessed the utility of a direct-to-consumer approach to executing pet microbiome studies. We characterized the gut microbiomes of 238 pets (46 cats and 192 dogs) by generating ~11 million merged reads that were mapped to the V4 region of 16S ribosomal RNA gene at a sequencing depth of 45,806 (±22,325) reads per sample. Analyses of these reads revealed that both feline and canine gut microbiomes are dominated by three major phyla, namely *Firmicutes*, *Proteobacteria*, and *Bacteroides* and that alpha diversity is higher in the feline gut. In addition to interspecies differences between the feline and canine gut, we also detected appreciable intraspecies bacterial variation within the canine population. While the dogs in this dataset could be assigned to three distinct clusters based on their gut microbiome, no clustering was observed within the feline population. Integration of additional data obtained from survey questionnaires revealed that geography and body weight may be associated with canine gut microbiome composition. Furthermore, we found that both the inter and intraspecies differences are more pronounced at finer taxonomic levels, indicating that strain-level investigations may be necessary in the future. This study demonstrates that the direct-to-consumer approach overcomes existing limitations in pet microbiome research, for example, it allows collection of large numbers of pet samples. The direct-to-consumer approach has proven successful in human genomics as well as human microbiomics and this study demonstrates that by building partnerships with an engaged general public this approach can also propel the field of pet microbiomics forward.

## Introduction

The mammalian gut harbors a myriad of microorganisms that are collectively known as the microbiota. A catalogue of microbes and their entire genomic contents in a particular environment is known as the microbiome. Recent studies in humans have demonstrated that the gut microbiome is influenced by lifestyle, likely due to differences in diet and environment associated with human subsistence [1–4] and that it plays an important role in health and disease [5–7]. There are many similarities between human and pet microbiomes [8] and emerging evidence suggests that gut microbiota may also act as an important regulator of health and diseases in household pets [9–12]. For example, antibiotics as well as dietary ingredients that vary between commercial foods have been shown to influence gut microbiomes in pets [10,13]. Moreover, gut microbial imbalances (dysbiosis) in pets can result in decreased production of short-chain fatty acids, possibly contributing to the development of chronic inflammatory conditions [14].

Pet health is an important and emerging field of research. Dogs and cats collectively number 135 million and total annual veterinary expenditure exceeds $17B in the US [15]. Increased understanding of pet microbiomes has the potential to illuminate novel aspects of host physiology and disease etiology that may lead to the development of therapeutics, including but not limited to dietary supplements, probiotics, and postbiotics [16–19]. Existing studies of pet microbiomes have several limitations. First, many studies tend to have limited sample sizes (<10 animals) [10,13,16,20–34]. Second, methodologies used to characterize and quantify gut microbial populations vary significantly between studies. Traditionally, bacteria were isolated from intestinal or fecal samples using culture-based methods [16–21,35–40], which introduce bias since they are not reflective of the host gut environment [10,41,42]. Finally, a wide variety of molecular methods of microbial characterization, ranging from denatured gradient gel electrophoresis (DGGE) [13,36,43], fluorescence in situ hybridization (FISH) [26,37,43], terminal restriction fragment length polymorphism (T-RFLP) [44], and 16S ribosomal RNA gene (16S rDNA) gene-based sequencing [13,16,17,21,22,38,45,46], have been employed in pet samples. Next-generation sequencing appears promising [8,23,24,27,28,30–34,47–74], but differences in sequencing platforms, extraction methods, and library preparation workflows have produced inconsistent results [75]. Due to the technological differences it has been difficult to generate meaningful insights by pooling samples across experiments.

In this proof-of-concept study, we assess whether a direct-to-consumer (DTC) approach can be used to overcome low sample sizes reported in previous pet microbiome studies. We assembled a sample size of 238 household pets comprising 46 cats and 192 dogs living in the United States, making this one of the largest pet microbiome studies to date. We used a culture-independent approach to estimate relative abundances of gut bacterial populations by generating high-coverage Illumina-based sequencing of V4 region of the 16S rDNA segments of these samples using standardized methods in microbial sequencing [76]. Using statistical and machine learning approaches, we characterize the canine and feline gut microbiomes and highlight inter- and intraspecies differences. Finally, we integrate additional data obtained using survey questionnaires from a subset of participants to identify factors associated with the canine and feline gut microbiomes.

## Materials and methods

### Ethics statement

This is an observational study carried out using noninvasive procedures from pet owners who voluntarily donated pet fecal matter. Customers consented to the use of generated non-identifying data for scientific study through the use of an electronic customer consent form.

### Animals and sample collection

Fecal samples were acquired from a total of 238 pets, including 192 dogs and 46 cats. NomNomNow customers collected stool samples using the NomNomNow Insights Microbiome Testing kit following instructions provided in the kit. Customers were instructed to collect pea-sized amounts of fresh feces (approximately 500 mg) in a collection tube containing a stabilizing buffer using sterile swabs provided with the kit. Collection tubes were shipped at room temperature to Laragen, Inc. (Culver City, California) where samples were stored at -20°C until further processing. Customers were asked to complete a brief online questionnaire including pet demographic and health questions at the time of registration. Only information to pets was gathered and none of the questions collected sensitive information about the pet owners. All pet-related information, including age, weight, and overweight status, were self-reported. The survey questions are included in the S1 File.

### DNA extractions, amplicon sequencing, and 16S variant analyses

DNA was extracted using a bead beating protocol by mixing 250uL of samples with 300 uL of lysis buffer (containing Tris, EDTA, NaCl, and SDS) with proteinase and Zirconium Beads (0.1mm and 0.5mm). The V4 region of the 16S rDNA gene was PCR amplified using the primers and protocols described previously by the Earth Microbiome Project [76]. The amplified DNA fragments were multiplexed and paired-end reads were generated using the Illumina MiSeq across 18 batches. We did not find disproportionate numbers of cats and dogs for any given batch (P = 0.11, *chi-squared test*). Paired-end reads across different batches were processed together using *DADA2* [77], and subsequently analyzed in R version 3.5.1 [78] using *phyloseq* [79] following best practices guides described previously [80]. In order to identify high quality sequences, reads were subjected to 10 base pairs trimming at each end. Sequences with ambiguous bases were removed and reads with more than two expected errors were discarded (trimLeft = c(10, 10), maxN = 0, maxEE = 2, truncQ = 2), and sequence variants were inferred by pooling reads from all samples (pool = TRUE). Amplicon Sequence Variant (ASV) tables were then created by merging paired-end reads and taxonomy was assigned using the RDP v14 training set [81].

Approximately 11.5 million merged reads were generated and 27,104 ASVs were initially identified. After quality control and chimera removal, 95% of the reads (depth = 10,902,218) and 5,052 taxa remained. Of these, a small fraction of taxa (31 ASVs) belonged to unknown bacterial phyla, and several additional phyla were present in less than 5% of samples, namely: Aquificae, Chlamydiae, Crenarchaeota, Deferribacteres, Euryarchaeota, Nitrospirae, Planctomycetes, Spirochaetes, SR1, and TM6. Removal of these taxa resulted in 10,901,731 reads and 5,007 taxa. After these quality control measures, the mean sampling depth per sample was 45,806 (SD±22,325). Each sample contained at least 13,679 reads and the maximum coverage was 94,183 reads. Hence, the final dataset included 10,901,731 reads belonging to 5,007 bacterial taxa in 238 samples.

### Statistical analyses

Two metrics of alpha diversity, namely species richness and Shannon diversity index, were computed by rarefying the samples to various depths starting from 1,000–13,500 sequences per sample by increasing the sequencing depth by 500 reads. One hundred iterations were performed at each depth and the mean values were used as the estimate of these measures in each sample. Kruskal–Wallis tests were used to assess the significance of differences in each of the alpha diversity metrics between the pet types at each rarefaction depth.

The nonrarefied 16S count data was log-transformed to calculate Bray-Curtis, Unifrac and weighted UniFrac distances using the *vegan* package [82] and Principal Coordinate Analyses (PCoA) were performed using all three distances. A generalized mixed-effects model was implemented to assess the differences in Bray-Curtis distances at each taxonomic level using the *lme4* package in R [83]. Batch and Labels (“Within Cats”, “Within Dogs”, “Between Cats and Dogs”) were used as fixed effects and each individual was treated as a random effect. Visual inspection of residual plots did not reveal any obvious deviations from homoscedasticity or normality at any taxonomic level. P-values were obtained by likelihood ratio tests of the full model (with Labels and Batch variables) against a null model (with only the Batch variable). *Permutational multivariate analysis of variance* (PERMANOVA) was performed using Bray-Curtis and UniFrac distances with 10,000 randomizations by including batch as one of the factors to assess the differences in community composition using the *vegan* package [82]. Differential abundance of bacterial taxa between cats and dogs was assessed at each taxonomic level using a negative binomial *generalized linear model* (GLM) using the *differential expression analysis for sequence count data version 2* (*DESeq2*) package in R [84]. Taxa with absolute log2 (fold change)>1.5 and FDR adjusted P-values<0.01 were considered significant. These analyses were repeated using the canine samples in the dataset to characterize gut bacterial variation within the canines. We also repeated the PCoA analysis using only the feline samples and observed no obvious intraspecies differences.

In order to identify the factors associated with canine gut bacteria, we implemented a generalized linear mixed-effects model with a maximum likelihood fit using batch and nine additional variables obtained from the survey questionnaire as fixed effects and individuals as a random effect using the *nlme4* package in R [85]. Therefore, the ten variables used in the model included batch, gender, age, weight, overweight status, activity level, overall health status, acquisition method, residence location (rural, suburban, urban), and geographic region. Samples were aggregated into four geographic regions based on US Census Bureau designations (Northeast: CT, ME, MA, NH, RH, VT, NJ, NY, PA; Midwest: IL. IN, MI, OH, WI, IA, KS, MN, MO, NE, ND, SD; South: DE, FL, GA, MD, NC, SC, VI, DC, WV, AL, KN, MS, TN, AK, LA, OK, TX; West: AZ, CO, ID, MT, NV, NM, UT, WY, AK, CA, HI, OR, WA). P-values were obtained by likelihood ratio test of the full model against a null model (only the batch variable). Shannon diversity index at rarefaction depth of 12,500 was used for alpha diversity. These nine variables plus the batch were also used to perform a PERMANOVA with 10,000 permutations in cats and dogs separately.

### Random forests

All random forest classifiers [86] were constructed using the repeated k-fold cross validation and random search implemented in R-package *caret* [87]. The data was partitioned into training and validation sets containing 70% and 30% of the samples respectively and ASVs were used as the predictors. The models were trained by optimizing the tuning parameters using 10-fold cross validation repeated 3 times and accuracy was used to select the optimal model. The performance of the classifiers was assessed by generating area under the receiver operating characteristic curves (AUC) using the R-package *ROCR* [88]. Variable importances were calculated using the default *varImp* function in the *caret* package.

### Clustering

Partitioning Around Medoids (PAM) Clustering was performed using the *cluster* package [89]. Individuals were clustered into multiple clusters (K = 1 to 5) based on the top three PCoA dimensions obtained using Bray-Curtis distances. Goodness of clustering was assessed using a “gap” statistic with 1,000 bootstrapped replicates. We did not find skewed distribution of dog clusters across batches (P = 0.48, *chi-squared test*). Finally, a PERMANOVA with 10,000 permutations was used to assess separation between the clusters using the Bray-Curtis and UniFrac distances.

## Results

### Sample description

We provided a brief survey questionnaire including demographic and health questions to our customers and obtained answers from about half of the participants (n = 123; 30 out of 46 cat owners and 93 out of 192 dog owners). Based on these surveys, our dataset contained at least four dozen canine breeds and a dozen different feline breeds. Domestic shorthairs (n = 11) and mixed breed mongrels (n = 17) were the most common cats and dogs in our dataset. Females and males represented similar proportions of cats (F = 53%, M = 47%, S1 Table) and dogs (F = 48%, M = 52%). Mean age for cats and dogs were 6.6 (±4.97) and 5.3 (±3.17) years respectively. Ninety seven percent of cats and dogs were reported to be healthy overall by their owners, with no reported acute or chronic diseases apart from being overweight or obese. On average, the cats and dogs weighed 4.6 (±1.4) and 13.3 (±10.4) lbs respectively and about 80% of both cats as well as dogs were reported to be of ideal weight. Also, 60% and 73% of cats and dogs were reported to be average or very active. Half of these samples were from the western United States while 12%, 11%, and 23% were from the midwestern, northeastern, and southern regions of the country. A majority of these pets resided in suburban regions, while 14% and 29% were from rural and urban locations. About half of the pets were obtained from breeders, 31% were rescued or adopted and the rest (17%) were obtained from other sources.

### Firmicutes, Proteobacteria, and Bacteroides dominate the canine and feline gut

In order to characterize the gut bacterial composition of cats and dogs we used the Illumina MiSeq to sequence the V4 region of 16S rDNA obtained from a total of 238 fecal samples (46 cats and 192 dogs), with an average of 45,806 (SD±22,325) high-quality reads per sample (S1 Fig). Although we detected a total of 23 known phyla and some reads were assigned to unknown bacterial phyla, the unknown phyla and 10 known phyla were present in less than 5% of samples and were thus removed (S1 Fig). Of the remaining 13 phyla, Firmicutes and Proteobacteria were most abundant followed by Bacteroidetes and together comprised at least 90% of gut bacteria in both cats and dogs (S2 Table). Firmicutes, Proteobacteria, and Bacteroides constituted 38% (Q1 = 21%,Q3 = 59%), 37% (Q1 = 3%,Q3 = 62%), and 14% (Q1 = 4%,Q3 = 22%) of the gut bacteria in cats and they comprised 45% (Q1 = 22%,Q3 = 66%), 32% (Q1 = 3%,Q3 = 52%), and 15% (Q1 = 1%,Q3 = 23%) in dogs. Similar proportions of the 13 phyla were obtained even after standardizing the abundances to the median sequencing depth (S2 Table). Of the 13 phyla, the abundance of only Actinobacteria and Fusobacteria differed significantly between the canine and feline gut (P<0.01 and abs(log2FC)>1.5, *GLM*, S3 Table). Actinobacteria was higher in the feline gut and Fusobacteria was higher in the canine gut (S2 Fig).

### Diversity differences between pet types are more pronounced at finer taxonomic levels

**Fig 1:**
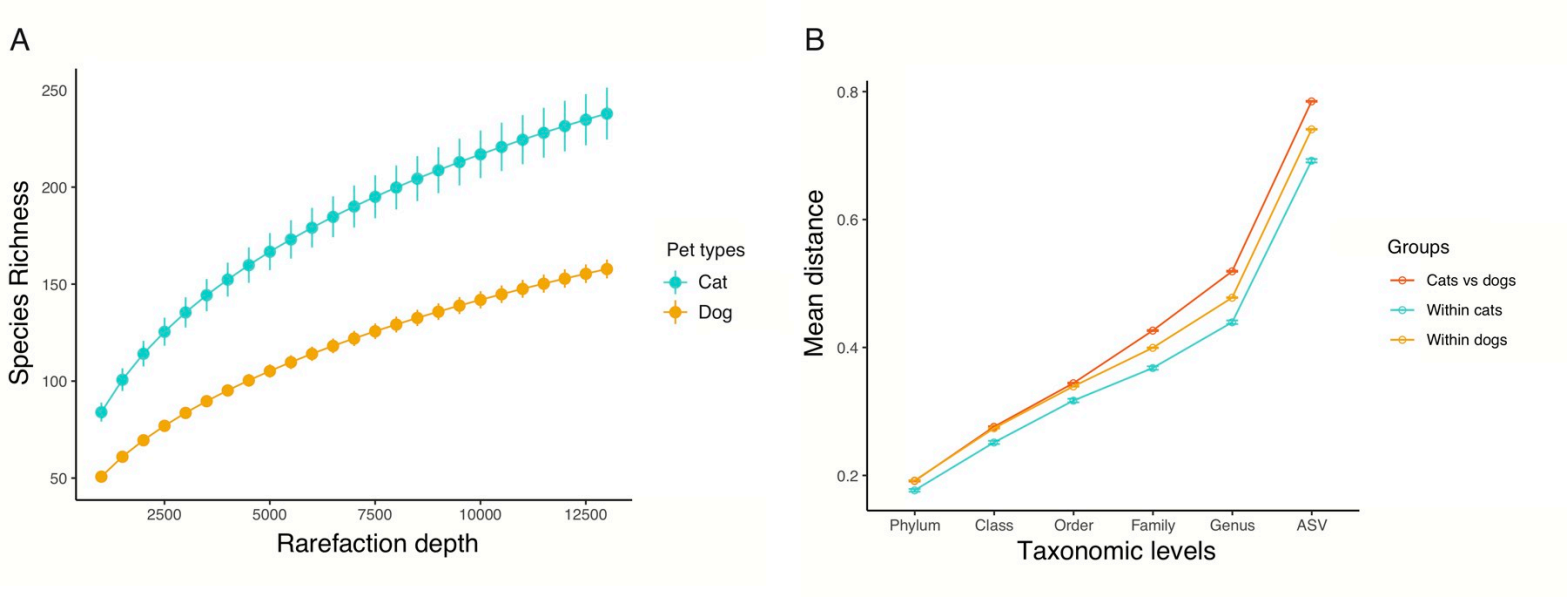
Comparison of bacterial diversity in the canine and feline gut. **(A)** Rarefaction curve showing alpha diversity differences between cats and dogs. Error bars represent standard deviations. **(B)** Bray-Curtis distances between cats and dogs (red), within cats (aquamarine), and within dogs (gold). Bars represent standard errors of the mean. The largest interspecies difference was observed at the ASV level and it was significantly larger than the two intraspecies distances.

Rarefaction curves generated using these reads revealed that the sequencing depth we achieved sufficiently saturated the bacterial diversity in both cats and dogs (Fig 1A). Next, we used two commonly used measures to characterize species diversity–species richness and Shannon diversity index–in the canine and feline gut. We computed canine and feline gut bacterial diversity at various rarefaction depths ranging from 1,000–13,500 reads. Both measures of gut bacterial diversity were higher in cats regardless of rarefaction depth (P<0.01, *Kruskal-Wallis test*, Fig 1A and S3A Fig), indicating that the feline gut harbors significantly higher bacterial diversity relative to the canine. This observation is consistent with previous reports [47,53].

Next, we evaluated differences in gut bacterial composition between cats and dogs using the Bray-Curtis distance at each taxonomic level. The interspecies distance was not visually different from the intraspecies distances at higher taxonomic levels (i.e., phylum, class, and order; Fig 1B). However, at finer taxonomic levels (i.e., family, genus, and ASV), the interspecies distance appeared to become significantly larger than the intraspecies distances. The difference between interspecies and intraspecies distances was the largest at the ASV level and did not level off, indicating that additional variation may exist at finer taxonomic details (strain or substrain level). Nevertheless, at the ASV level, the interspecies distance was significantly larger than both the intraspecies distances (FDR adjusted P<0.001, *Kruskal-Wallis* and *Dunn’s post-hoc test*). Thus, we conducted all subsequent analyses at the ASV level because it provided the highest resolution of gut bacterial diversity.

### Differences in overall bacterial composition between canine and feline guts

**Fig 2:**
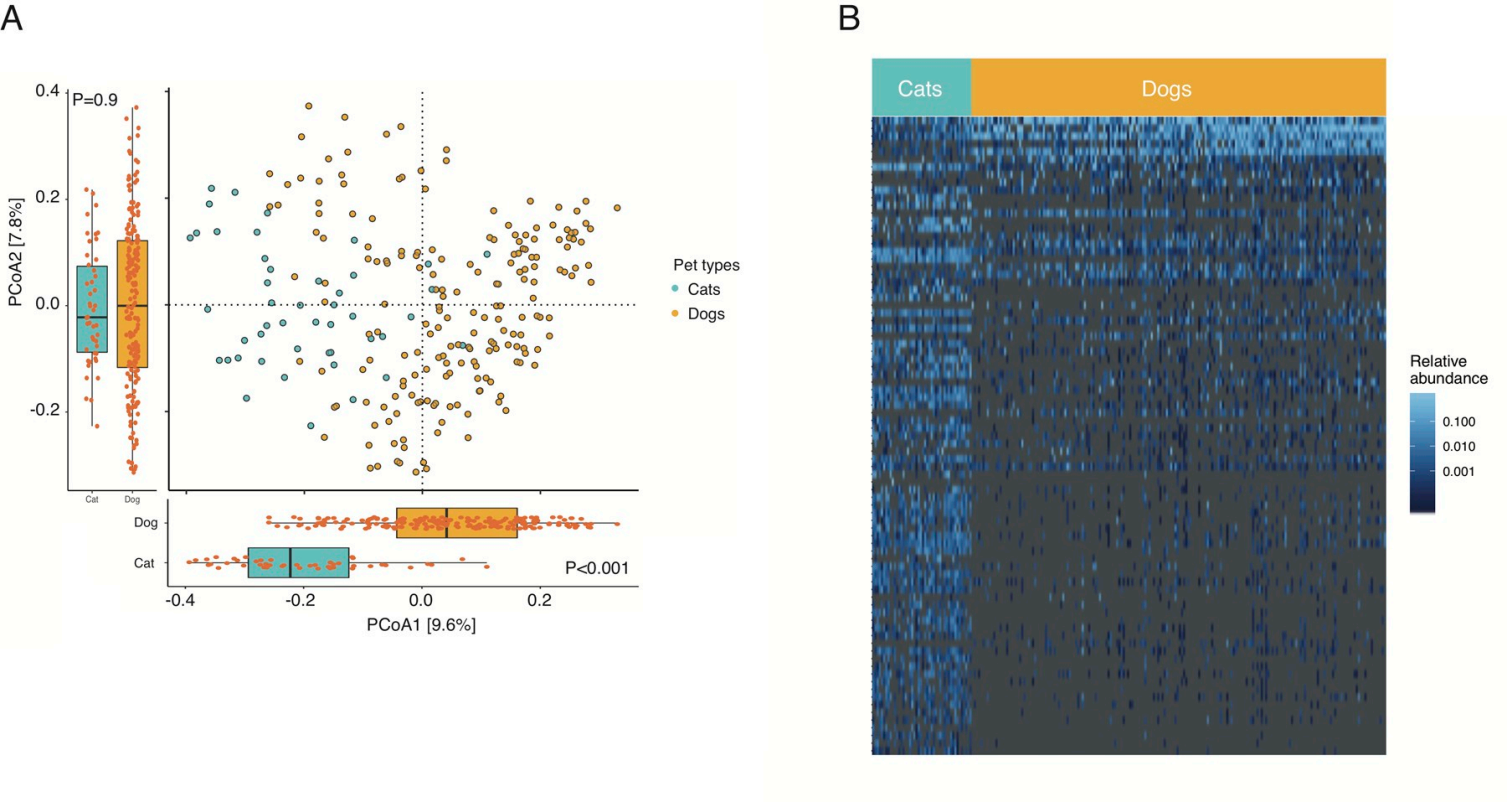
Gut microbiome variations between cats and dogs. **(A)** PCoA of Bray-Curtis distance reveals separation between the gut microbiomes of cats and dogs at the ASV level. Box plots highlight the interspecies differences along the PCoA1. Dots represent individuals and colors indicate species. **(B)** Heatmap displaying 83 taxa with significantly different abundance between the canine and feline populations with individuals in columns and taxa in rows. Colors represent relative abundances. The 83 taxa included in this heat plot are listed in S5 Table.

A Principal Coordinates Analysis (PCoA) revealed distinctions between the feline and canine gut bacteria (Fig 2A). To evaluate whether such difference could be due to batch effect or could be highlighting biological differences between the canine and feline gut bacterial compositions, we performed a PERMANOVA with two variables–batch and pet types (in this order). The samples in this study were sequenced across 18 different batches and a significant contribution of batch effect was detected (P<0.001, *PERMANOVA*). However, after accounting for batch effects, we observed significant differences between pet species and gut bacterial composition (P<0.001 for batch effects as well as pet species, *PERMANOVA*, Fig 2A). The first principal coordinate axis (PCoA1) clearly separated the cats from the dogs (P<0.001, *Wilcoxon-rank sum test)*. No significant differences were observed between cats and dogs along the PCoA2 (P = 0.9, *Wilcoxon-rank sum test)*. Interestingly, an appreciable number of dogs lay at an intermediate position along the PCoA1 and were separated from the rest of the dogs along the PCoA2. Hence, the PCoA1 seems to primarily underscore the interspecies differences while the PCoA2 appears to highlight intraspecies variations within the canine gut microbiome. These findings were consistent using the UniFrac and weighted UniFrac distances as well (S4 Fig).

We also used machine learning (random forest) as an alternative approach to assess differences between the canine and feline gut bacterial compositions. A Random Forest classifier was able to categorize the individuals to their respective species with high accuracy (accuracy = 96%; 95%CI = 88%, 99%; AUC = 0.99, S3B Fig and S4 Table). Over 96% of dogs (N = 132 out of 134 in the training data and 57 out of 59 in the validation set) were correctly classified as dogs and over 81% of cats (N = 26 out of 32 in the training data and 12 out of 13 in the validation set) were also correctly identified as cats (S4 Table). Therefore, these findings collectively demonstrate that the bacterial compositions of canine and feline gut differ significantly.

### Gut bacterial taxa differences between canine and feline samples

Next, we sought to identify bacterial taxa that differentiate the canine and feline populations. To do so, we compared the abundance differences of the 5,007 ASVs between cats and dogs using DESeq2 [84]. We found 238 taxa with significantly different abundance between the two species (FDR adjusted P<0.01 and absolute fold change>1.5, *GLM*, S3 Table). Many of the significant taxa identified using this approach also had higher variable importance in the random forest model (S3C Fig). A total of 83 taxa exceeded both significance criteria in the DESeq analyses and were among the top 10% of taxa with high variable importance in the random forest analysis. As a conservative estimate, we considered these 83 taxa as differentially abundant between cats and dogs (Fig 2B). Bacteria belonging to the genera *Enterococcus*, *Fusobacterium*, *Megamonas* and *SMB53* were elevated in dogs relative to cats while members of *Adlercreutzia*, *Alistipes*, *Bifidobacterium*, *Carnobacterium*, *Collinsella*, *Coprococcus*, *Desulfovibrio*, *Faecalibacterium*, *Oscillospira*, *Parabacteroides*, *Peptococcus*, *Peptostreptococcus*, *Ruminococcus*, *Slackia*, and *Sutterella* were relatively enriched in cats. Although several members (ASVs) of the genera *Bacteroides*, *Blautia*, *Clostridium*, and *Dorea* were differentially abundant between the two species, some members from these genera were enriched in cats and others were found at higher abundance in dogs (S5 Table).

### Gut bacterial variations within the canine and feline samples

**Fig 3:**
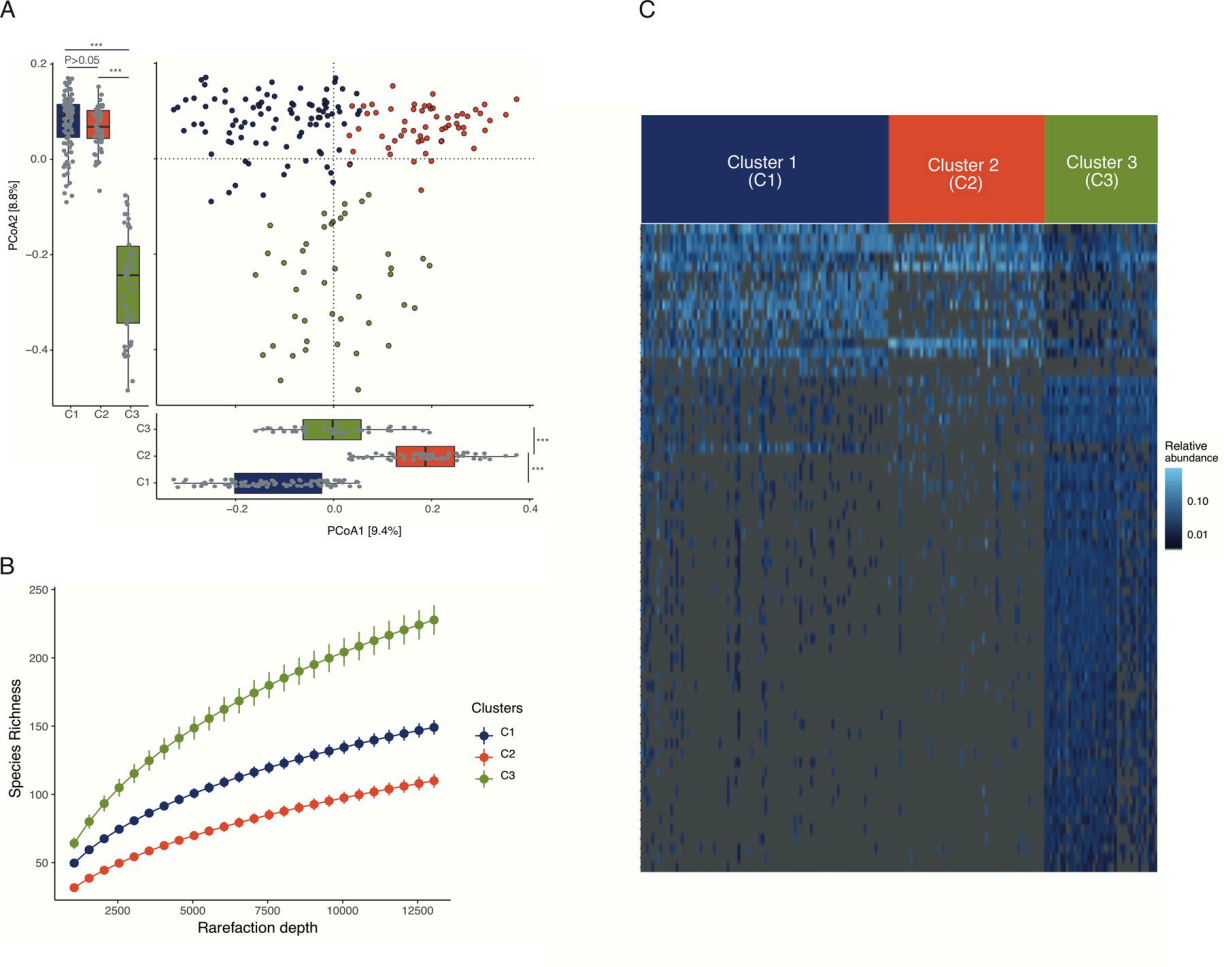
Gut microbiome variations within dogs. **(A)** PCoA analysis using Bray-Curtis distances revealed significant variations in the canine gut microbiome at the ASV level. All three clusters were significantly different along PCoA1 but only Cluster 3 differed significantly along PCoA2. *** indicates P<0.001 in both *Kruskal-Wallis and Dunn’s post-hoc* tests. **(B)** Alpha diversity measured using species richness also varies significantly between the three canine clusters. **(C)** Heatmap displaying 68 ASV with significantly different abundance between the three canine clusters with individuals in columns and ASV in rows. Colors represent relative abundances. The 68 taxa included in this heat plot are listed in S5 Table.

The previous analyses investigating interspecies differences in gut bacterial composition and taxa abundances (Fig 2) also indicated that there may be appreciable microbial variation within dogs. To test this hypothesis, we first performed a PCoA exclusively on the 192 dogs in our dataset, which revealed clear structure (Fig 3A). To determine the optimal number of canine groups, we performed a cluster by ordination analysis using the top three PCoA axes (S5A Fig) [80]. Goodness of clustering assessed using a “gap” statistic revealed three canine clusters (Clusters I,II, and III; S5B Fig). After accounting for batch effects, the gut bacterial composition differed significantly between the three clusters (P<0.001 for batch effects as well as clusters, *PERMANOVA*, Fig 3A). Next, we calculated distances between individuals within and across groups as described above. For all three clusters, within-cluster distances were significantly smaller than the between-cluster distances (FDR adjusted P<0.001, *Kruskal-Wallis* and *Dunn’s post-hoc tests*, S5C Fig). We also used random forest to assess whether the individuals can be accurately classified into their respective groups based on their ASV counts. Overall accuracy of the random forest model was 92% (95%CI = 0.8102, 0.9714) (S5D Fig) and classification accuracies for Clusters I, II, and III were 95% (AUC = 0.996), 93% (AUC = 0.997), and 87% (AUC = 0.986) respectively. Finally, we used species richness to compare alpha diversity between these three clusters and found significant differences between the three clusters at all rarefaction depths (P<0.001, *Kruskal-Wallis* and *Dunn’s post-hoc tests*, S5C Fig). These analyses strongly support that the gut microbiome varies significantly between the canine clusters.

To assess whether the cats in this study can be assigned to distinct clusters based on their gut microbiomes, we performed a cluster by ordination analysis using the 48 feline samples in our dataset. Neither the PCoA nor the gap statistics revealed clear structure within cats (S6 Fig).

In order to identify bacterial taxa that differentiate the canine clusters, we compared the abundance differences of the 4,449 ASVs present in the dogs using DESeq2 as well as a random forest model as described above. We found 68 taxa with significantly different abundances among the three canine clusters (FDR adjusted P<0.01, absolute fold change>1.5, *GLM*, and top 10% tail of variable importance in random forest analysis, Fig 3C). The vast majority of bacteria distinguishing the canine clusters belonged to the genus *Bacteroides* (80%, N = 55 out of 68, S6 Table).

### Factors associated with canine gut bacterial variations

**Fig 4:**
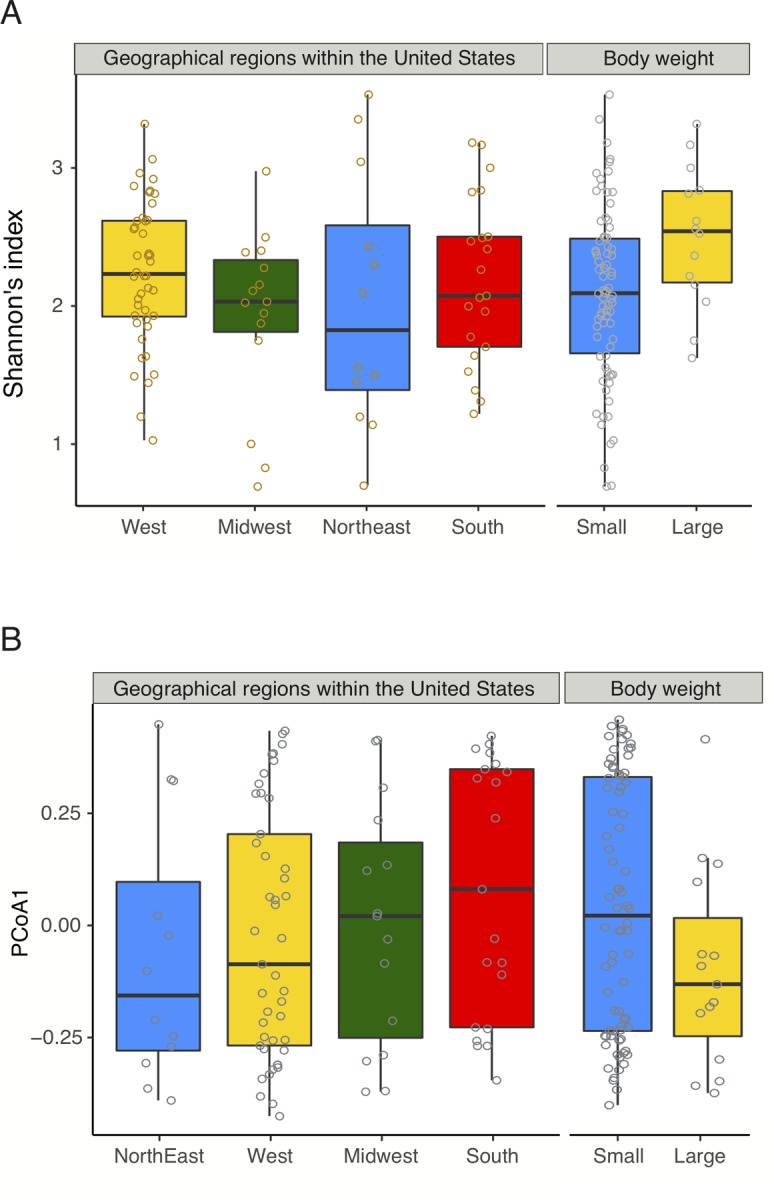
Factors associated with canine gut microbiome variations. **(A)** Alpha diversity differences between dogs residing in different geographical regions within the United States (left) and between large and small dogs (right). **(B)** Canine beta diversity differences across the USA and between large and small dogs along the PCoA1 obtained from 92 dogs for which additional data from survey questionnaires were available.

Next, we assessed whether gut bacterial diversity and composition were associated with any of the factors collected via the survey questionnaires, namely gender, age, weight, overweight status, overall health status, activity level, mode of acquisition, household environment (rural, suburban, and urban), and region of residence within the United States (West, Midwest, Northeast, and South). Survey data were available from 30 cats and 93 dogs; thus, we performed subsequent analyses by focusing on the dogs. One of the dogs was an obvious outlier with very high Shannon diversity index; hence, we removed it and evaluated associations between the nine factors listed above (using batch as an additional variable) and alpha diversity as well as gut microbiome composition on the remaining 92 dogs. We observed significant associations between canine alpha diversity and geography as dogs from the Western United States had higher Shannon diversity index compared to the Midwestern dogs (P = 0.009, *GLM*, Fig 4A), although differences between the Western dogs and Northeastern or Southern dogs were not significant. Similarly, compared to the rural dogs, suburban dogs had lower Shannon diversity index (P = 0.039, *GLM*), although no noticeable differences were observed between rural and urban dogs. Interestingly, the association between body weight and Shannon diversity index approached significance in dogs (P = 0.097, *GLM*), with Shannon diversity index values significantly higher in larger dogs (over 22.7kg) relative to smaller dogs. Furthermore, gut microbiome composition was also associated with geographic region and its association with body weight approached significance (P = 0.017 and 0.075, *PERMANOVA*, Fig 4B). None of the other variables, including gender, age, overweight status, and household environment (rural, suburban, and urban) were significantly associated with gut microbiome composition in this canine population.

## Discussion

Companion animal microbiome research has historically suffered from several limitations. First, purchasing and housing large numbers of dogs and cats in the laboratory can be expensive. Thus, pet studies have historically suffered from poor statistical power due to small sample sizes leading to inconsistencies in findings across studies. Second, research designs that confine pets to the laboratory raise ethical concerns. Third, the limited biological and environmental diversity generally represented in laboratory samples compromises the generalizability of findings to the broader and arguably more diverse companion animal population. Finally, while several large scale cohorts have been established to study human microbiomes [90,91], funding for large scale pet cohorts is lacking [10]. Using a DTC approach for sampling backed by industry allowed us to circumvent many of the obstacles listed above. By engaging pet owners directly, we eliminated the need to purchase and house animals in the laboratory. We not only sampled a relatively large number of dogs and cats but we were also able to sequence the gut microbiomes at much deeper coverage than many of the previously published studies. Our sample size of 195 dogs and 48 cats sequenced at an average depth of 45,547 reads makes this one of the largest pet microbiome studies to date. Additionally, because these heterogeneous samples were collected directly from consumers, they may more accurately represent the naturally occurring microbiomes of household pets.

Many of our results are in congruence with previous studies conducted in academic laboratories. We found that Firmicutes, Proteobacteria, and Bacteroides were the most prevalent bacteria in both the feline and canine gut microbiomes, which is consistent with previous studies [22–24,32,92,93]. Firmicutes, Proteobacteria, and Bacteroides constituted 45% (Q1 = 22%,Q3 = 66%), 32% (Q1 = 3%,Q3 = 52%), and 15% (Q1 = 1%,Q3 = 23%) of the gut bacteria in dogs and they comprised 38% (Q1 = 21%,Q3 = 59%), 37% (Q1 = 3%,Q3 = 62%), and 14% (Q1 = 4%,Q3 = 22%) in cats, also comparable to previously-reported proportions. [22–24,32,92,93]. Comparative analyses demonstrated that the bacterial compositions of canine and feline guts differ significantly. We found significantly higher alpha diversity in cats relative to dogs, which also corroborates earlier findings [47,53]. Therefore, together these findings reveal that the DTC approach implemented herein produces results comparable to those obtained using standard techniques in the laboratory.

In addition to replicating previous findings, this study revealed novel insights into pet gut microbiomes. Numerous studies have shown that both diet and environment may influence the gut microbiomes and it is possible that species cohabiting a shared environment may have high degrees of similarities in gut microbiomes [1,3,8]. Our results suggest that despite the common environment, the gut microbiomes of dogs and cats differ significantly from one another. Alpha diversity was higher in cats relative to dogs, although the drivers of this difference remain to be identified. While dietary and behavioral differences are the usual suspects, differences in gut volume may also be driving the gut microbial differences between these pets [94,95]. Moreover, we observed the largest interspecies differences at the ASV level without leveling off, indicating that small genetic variations within bacterial populations, such as those observed between bacterial strains or substrains, differentiate canine and feline samples in this study.

In addition to the observed interspecies differences, our analyses strongly support the presence of appreciable levels of gut bacterial variations (both alpha and beta diversity) in dogs in this study. Based on their gut microbiomes, we were able to detect three distinct clusters of dogs. Curiously, we did not observe gut microbiome-based clustering in cats. The lack of clustering in cats may be due to smaller sample size of feline participants in this study. Alternatively, large differences in canine gut volume may be contributing to pronounced differences in gut microbial variations within dogs [94,95], while lack of marked variation in feline body sizes may be contributing to the lack of clustering in cats. Additional work is needed to pinpoint the drivers of such clustering in dogs and their biological relevance.

Although additional data was available from a limited number of participants, by integrating the survey data, we observed that gut microbial variations are significantly associated with body weight and geographic location. We found slightly higher bacterial diversity in larger dogs, which may be due to differences in canine gut volume and/or a more developed cecum and colon as well as a slower transit time of possible substrates for bacterial fermentation [95]. We also observed differences in the gut microbiomes (both alpha and beta diversity) of dogs from the Western and Midwestern parts of the United States. None of the other variables, including gender, age, and overweight status were significantly associated with the gut bacterial variations (both alpha and beta diversity) in this canine population. Previous studies have shown strong correlations between gut microbiome and urbanization in humans [1,2], although correlations between urbanization gradient and shifts in gut microbiome were not observed in dogs in this dataset. This could possibly be due to homogeneity in pet food across the United States, with most pets being on a commercially prepared kibble diet. Alternatively, the sample size of the rural dogs in our dataset may be too small to delineate the effect of urbanization in this dataset. It is noteworthy that these analyses were conducted in a subset of study individuals for which we had survey data, and we were unable to robustly assess associations between any of these factors and the feline gut microbiome due to the small number of cat samples in our study. In addition, the survey questionnaires we used captured limited information from participating animals. Therefore, the absence of association of particular traits (eg. breed, age, or sex) with the gut microbiome may simply underscore the limitations of this dataset. Collecting more detailed information using improved survey questionnaires and integrating them with larger sample sizes in the future will enable identification of additional factors associated with canine and feline gut microbiomes.

Our results also suggest that characterization of bacterial variation at finer taxonomic levels in the canine and feline gut may reveal additional inter- as well as intraspecies differences. Strain or substrain level variations may also be biologically relevant; for example, a previous study demonstrated that a specific strain of bacteria, *Bifidobacterium animalis* AHC7, prevents pathogens from colonizing the canine gut [18]. In addition to the differences in bacterial abundance, differences in functional categories can also be important indicators of gut health [8,12,63,69,73]. However, 16S is not useful in resolving taxonomic differences beyond genus level and it has limited utility in assessing functional profiles of the gut microbiome. Hence, we recommend that future microbiome studies include metagenomics approaches that generate strain level data using high throughput sequencing methodologies such as shotgun sequencing, with methods which would ideally be consistent across studies. In addition to providing strain level information, metagenomic approaches also have the potential to reveal functional differences (e.g., those obtained via KEGG module analysis) that provide a deeper understanding of the functions of the gut microbiome. A handful of studies have already implemented a shotgun metagenomics approach to delineate the role of microbiome in animal health and diseases [8,12,63,69,73]. If deep shotgun sequencing is prohibitively expensive, shallow-depth shotgun sequencing may be alternative to 16S sequencing as it can provide species and/or strain level taxonomic resolution as well as functional data at approximately the same cost as 16S sequencing [96].

Since the DTC approach can circumvent many of the obstacles in companion animal studies, it can be highly useful in studying the effect of pet microbiomes in health and disease in the future. Larger cohort sizes assembled using a DTC approach ideally will be able link microbiomes with specific health-related traits in pets. Identification of trait-specific microbial strains, functions, and metabolites can inform the development of new pet health products (e.g. probiotics) and treatments (e.g. microbial medicines, postbiotics) in pets [16,97]. Furthermore, given these pets share a common household environment with humans and have common gut bacteria, a better understanding of pet microbiomes has the potential to illuminate novel links between the microbiome and human diseases [8,12,63,69,97–100].

Despite the demonstrated utility of our approach, there are several limitations of this study. First, the 16S rDNA sequencing performed herein provides limited taxonomic resolution and does not fully capture the bacterial diversity in the canine and feline gut. Emerging evidence also demonstrates that other microbes, such as viruses, archaea, and yeast, cohabit and interact with bacteria in the gastrointestinal tract and may contribute to canine and feline health and diseases [24,101–103]. These microbes were not evaluated given the sequencing methods employed. Samples obtained from cats were fewer than those from dogs, which limited the ability to potentially identify clustering in these feline samples. Environmental variables, especially diet, varied between dogs and cats in this investigation and were not controlled. Elimination of confounding variables via controlled studies might reduce heterogeneity in the samples, however this would not represent the inherent diversity in the pet population. Integration of demographic, medical, nutritional, and lifestyle data through concurrent health assessments would increase understanding of gut microbial variations within these animals. We are working towards addressing these limitations in the future, with the ultimate goal of developing microbiome-based interventions for preventing and treating disease in pets.

## Conclusions

This proof-of-concept study shows that the DTC approach to studying pet microbiomes circumvents many of the challenges present in veterinary research, while producing comparable findings as well as novel insights. Using 16S rDNA sequencing of 192 dogs and 46 cats, not only do we show gut microbiome differences between these two species but we also demonstrate appreciable gut microbiome variations within the sampled dogs, and that geography and body weight may be associated with canine gut microbiome diversity as well as composition. Despite these findings, insights on pet microbiomes gained from 16S rDNA are limited and we recommend that future studies explore metagenomics approaches that can provide strain level data as well as functional characteristics of the gut bacteria.

## Supporting information

S1 Fig Quality control of the 16S sequencing data. **(A)** Total number of reads in the raw data. **(B)** Initially we identified 23 known phyla in the dataset as well as some bacteria that could not be assigned to known phyla based on their 16S rDNA. Many of these phyla were covered by few reads and were only present in less than 5% of study samples and therefore were removed. **(C)** Removal of such lowly abundant taxa did not result in significant loss in sequencing depths. **(D)** Prevalence of the 13 remaining phyla across individuals (y-axis) and their abundance in the dataset (x-axis).
(PNG)

S2 Fig Phylum level differences between cats and dogs. Relative abundance of Actinobacteria was higher in the feline gut and Fusobacteria was higher in the canine gut.
(TIFF)

S3 Fig Comparison of canine and feline gut microbiomes. **(A)** Rarefaction curve showing alpha diversity differences between cats and dogs using Shannon diversity index. Error bars represent standard deviations. **(B)** Performance of the random forest classifier (at the ASV level) assessed using ROC curves (cat: turquoise, dog: orange). AUC = 0.99 for both. **(C)** Identification of differentially abundant ASVs using log2 fold-change and variable importance factor (scaled to 100).
(PNG)

S4 Fig Comparison of gut microbiomes using UniFrac distances. **(A-B)** Comparison of canine and feline gut microbiome using UniFrac distance (top) and weighted UniFrac distance (bottom). **(C-D)** Comparison of gut microbiome across the three canine clusters using UniFrac distance (top) and weighted UniFrac distance (bottom). All P-values were obtained from PERMANOVA using 10,000 permutations.
(TIFF)

S5 Fig Intraspecies variations within the canine gut microbiome. **(A)** Scree plot showing the eigenvalues for the first 20 PCoA axes. **(B)** Gap statistic obtained from PAM clustering using the top 3 PCoA axes and 1,000 bootstrapped replicates. **(C)** Bray-Curtis distances within (white) and between (grey) the three feline clusters. In all three cases, the within cluster distances were smaller than the between group distances. **(D)** ROC curves evaluating the random forest classifier model to predict feline clusters.
(TIFF)

S6 Fig Intraspecies variations within the feline gut microbiome. Neither the PCoA analysis **(A)** nor the gap statistic obtained from PAM clustering **(B)** revealed distinct clusters in cats. We performed 1,000 bootstrapped replicates for clustering using the top 3 PCoA axes **(C)**.
(TIFF)

S1 Table Characteristics of pets in this study. (XLSX)

S2 Table Prevalence of bacterial phyla in canine and feline gut. (XLSX)

S3 Table Comparison of taxa abundance across taxonomic categories between canine and feline gut. (XLSX)

S4 Table Random forest analyses. (XLSX)

S5 Table Differentially abundant bacterial taxa (ASVs). Abundance differences between cats and dogs based on p-values and log2 fold change in DESeq and variable importance in the Random Forest analysis.
(XLSX)

S6 Table Differentially abundant bacterial taxa (ASVs) between different dog clusters. Abundance differences between canine clusters based on p-values and log2 fold change in DESeq and variable importance in the Random Forest analysis.
(XLSX)

S1 File Survey questionnaire. (PDF)

S2 File A phyloseq object containing ASV tables, phylogenetic trees, and sample data used in this study. (ZIP)
