# Supplementary figures and images for: Characterization of gut microbiomes of household pets in the United States using a direct-to-consumer approach
Source: PLoS One. 2020 Feb 20;15(2):e0227289. doi: 10.1371/journal.pone.0227289 (PMC7032713; doi:10.1371/journal.pone.0227289)

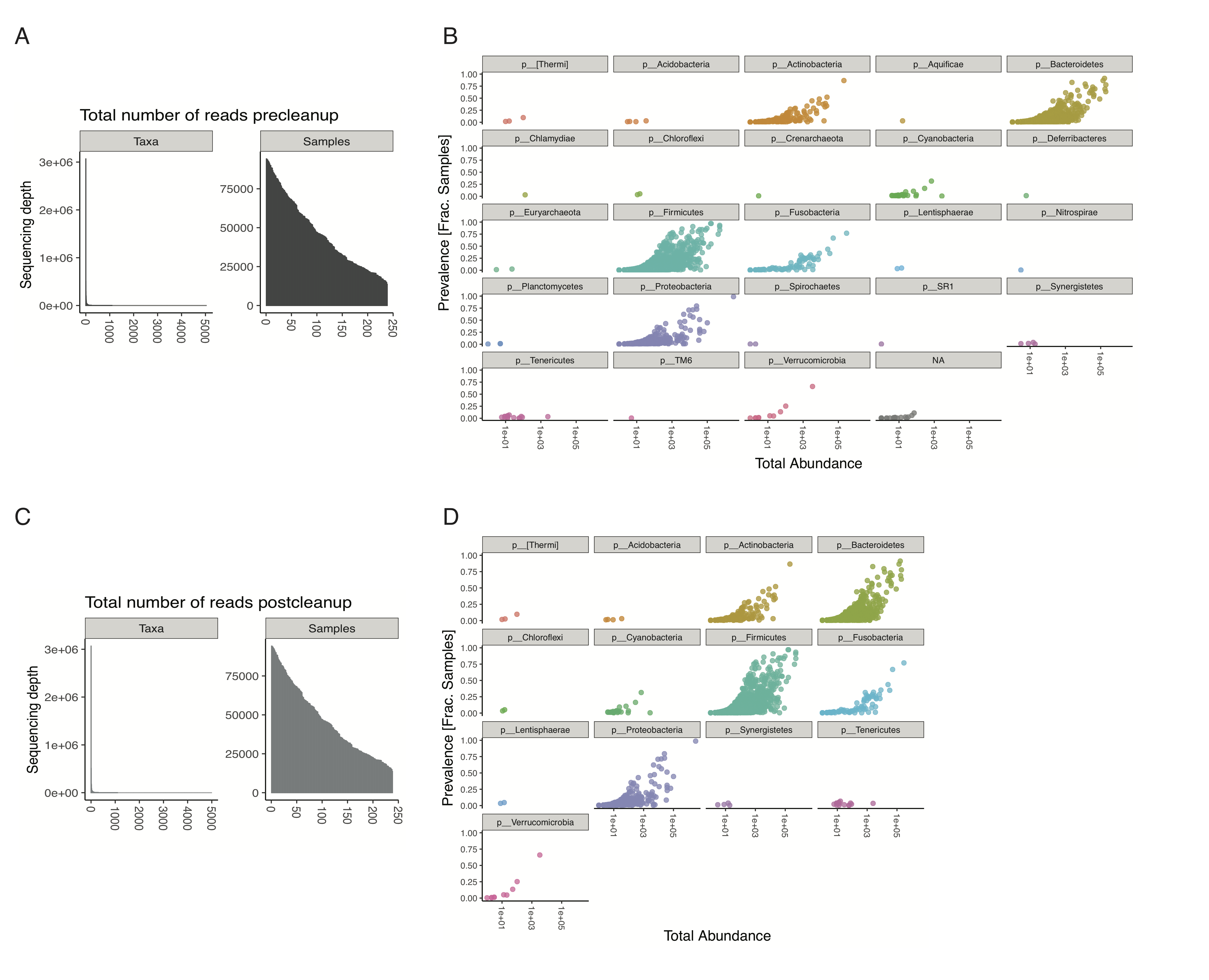

Supplement: S1 Fig — (A) Total number of reads in the raw data. (B) Initially we identified 23 known phyla in the dataset as well as some bacteria that could not be assigned to known phyla based on their 16S rDNA. Many of these phyla were covered by few reads and were only present in less than 5% of study samples and therefore were removed. (C) Removal of such lowly abundant taxa did not result in significant loss in sequencing depths. (D) Prevalence of the 13 remaining phyla across individuals (y-axis) and their abundance in the dataset (x-axis). (PNG) [file pone.0227289.s001.png]

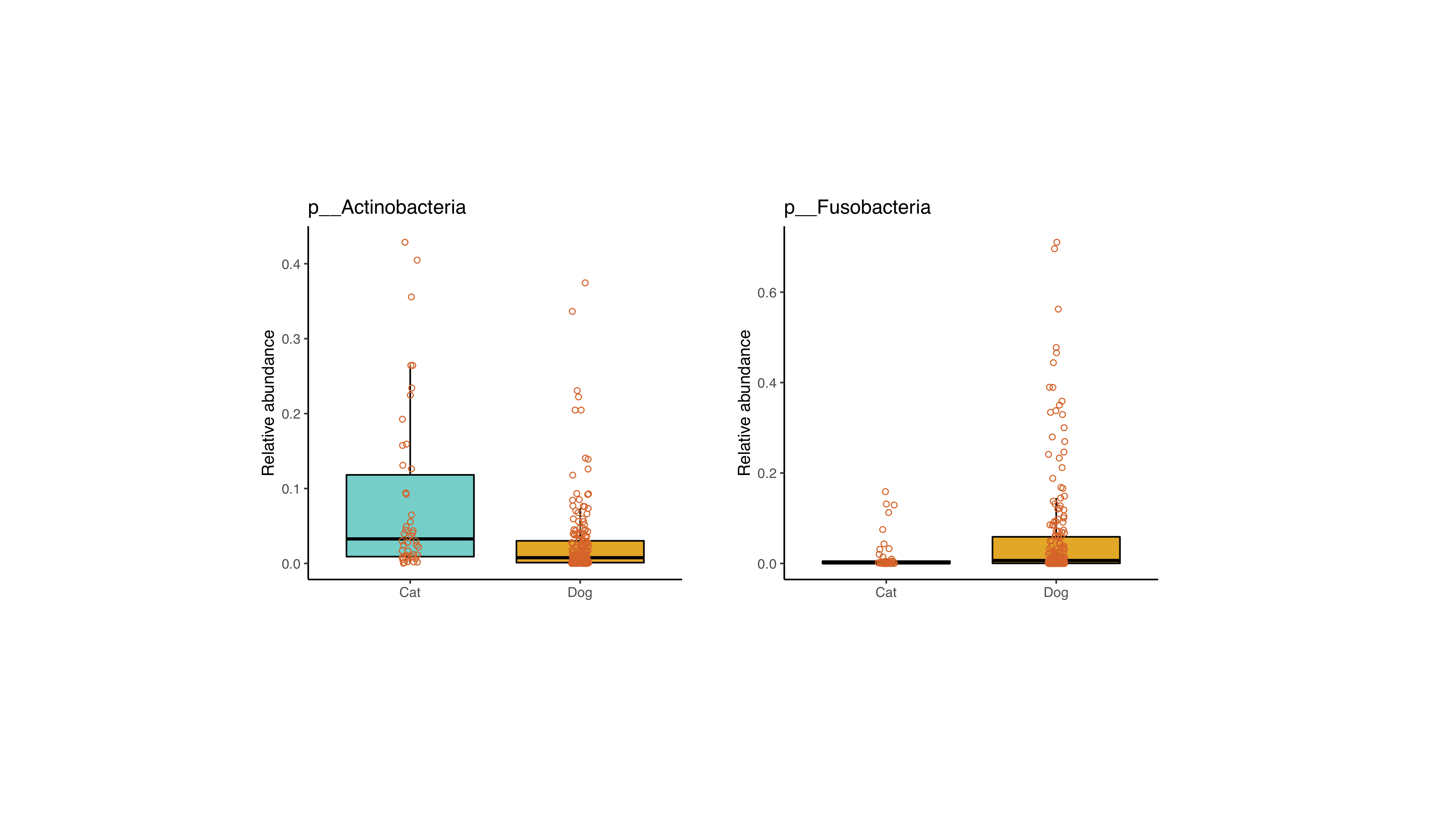

Supplement: S2 Fig — Relative abundance of Actinobacteria was higher in the feline gut and Fusobacteria was higher in the canine gut. (TIFF) [file pone.0227289.s002.tiff]

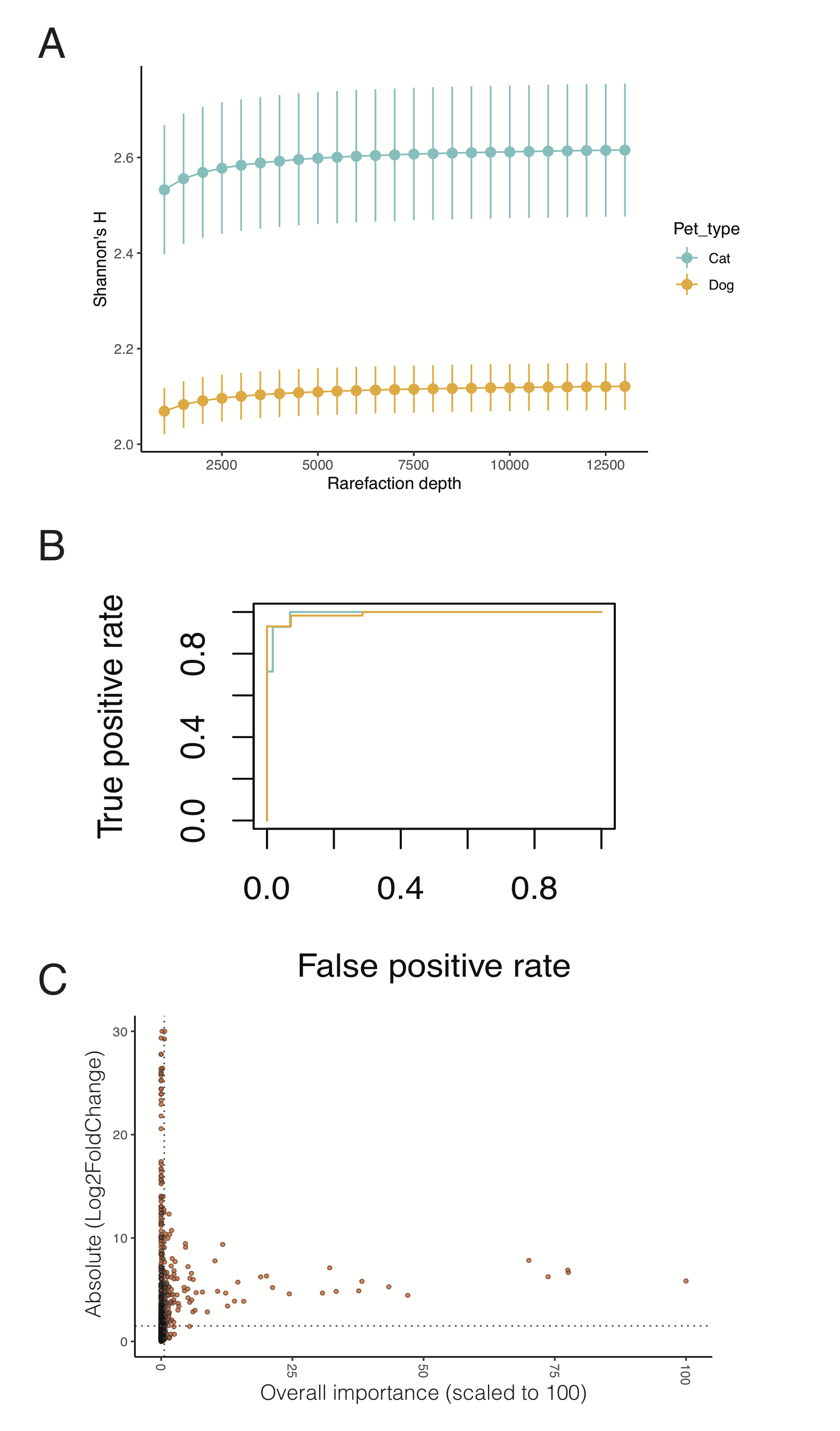

Supplement: S3 Fig — (A) Rarefaction curve showing alpha diversity differences between cats and dogs using Shannon diversity index. Error bars represent standard deviations. (B) Performance of the random forest classifier (at the ASV level) assessed using ROC curves (cat: turquoise, dog: orange). AUC = 0.99 for both. (C) Identification of differentially abundant ASVs using log2 fold-change and variable importance factor (scaled to 100). (PNG) [file pone.0227289.s003.png]

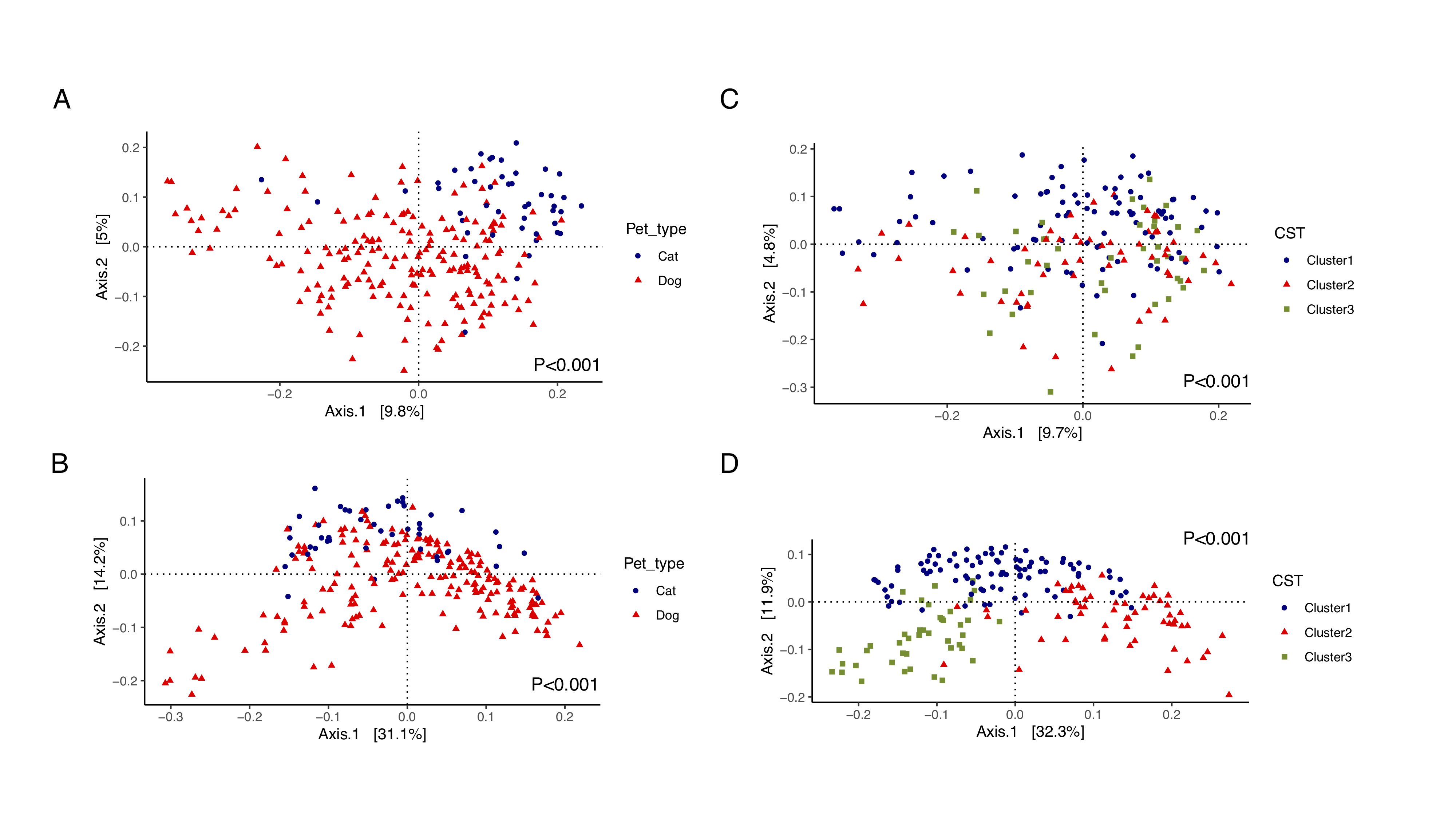

Supplement: S4 Fig — (A-B) Comparison of canine and feline gut microbiome using UniFrac distance (top) and weighted UniFrac distance (bottom). (C-D) Comparison of gut microbiome across the three canine clusters using UniFrac distance (top) and weighted UniFrac distance (bottom). All P-values were obtained from PERMANOVA using 10,000 permutations. (TIFF) [file pone.0227289.s004.tiff]

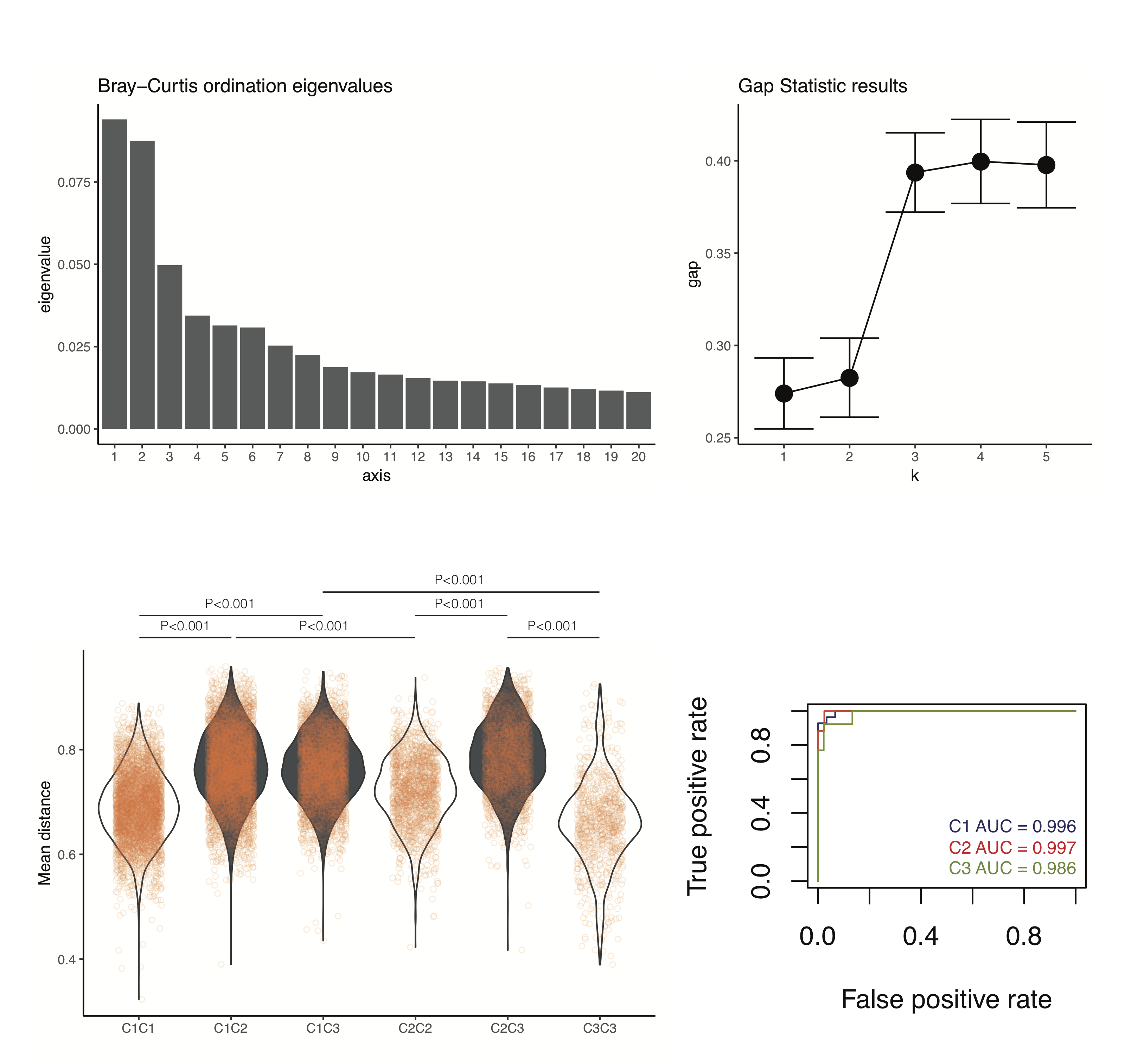

Supplement: S5 Fig — (A) Scree plot showing the eigenvalues for the first 20 PCoA axes. (B) Gap statistic obtained from PAM clustering using the top 3 PCoA axes and 1,000 bootstrapped replicates. (C) Bray-Curtis distances within (white) and between (grey) the three feline clusters. In all three cases, the within cluster distances were smaller than the between group distances. (D) ROC curves evaluating the random forest classifier model to predict feline clusters. (TIFF) [file pone.0227289.s005.tiff]

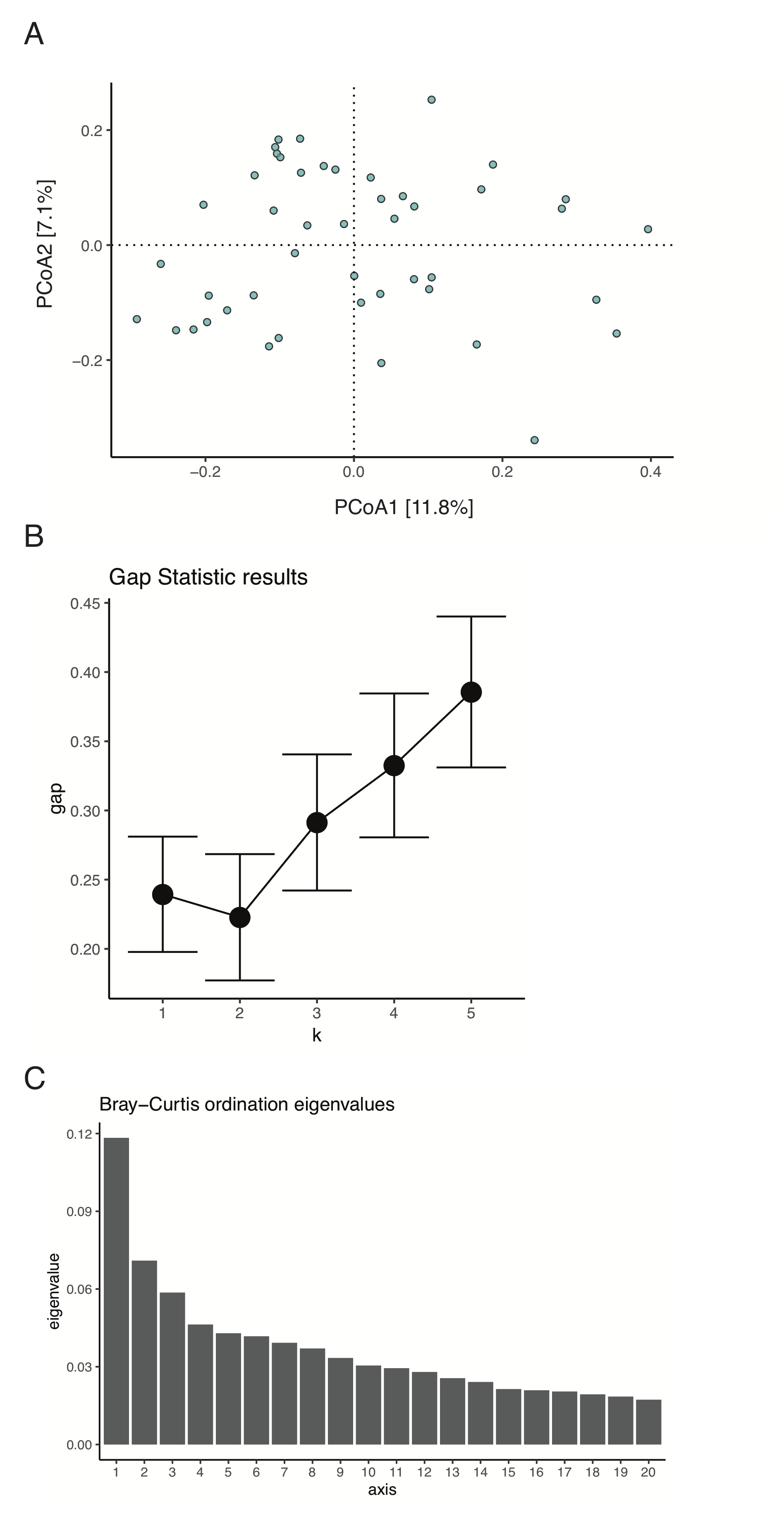

Supplement: S6 Fig — Neither the PCoA analysis (A) nor the gap statistic obtained from PAM clustering (B) revealed distinct clusters in cats. We performed 1,000 bootstrapped replicates for clustering using the top 3 PCoA axes (C). (TIFF) [file pone.0227289.s006.tiff]
