# Supplementary material for: Characterization of gut microbiomes of household pets in the United States using a direct-to-consumer approach
Source: PLoS One. 2020 Feb 20;15(2):e0227289. doi: 10.1371/journal.pone.0227289 (PMC7032713; doi:10.1371/journal.pone.0227289)
Supplement: S1 File — (PDF) [file pone.0227289.s013.pdf]

Survey questionnaire

SampleID: ______________________ Date: ______________________

Pet_type: ☐ Dog ☐ Cat Gender: ☐ Male ☐ Female

Pet's birthday: ______________________ Breed: ______________________

Is your dog a puppy? ☐ Yes ☐ No State of residence: ____________

Current weight of your pet (in lbs): ____________

Is your pet currently overweight? ☐ Yes ☐ No

Home environment: ☐ Rural ☐ Suburban ☐ Urban

How was the pet acquired? ☐ Breeder ☐ Rescue/Adoption ☐ Other

If other, specify: ____________________________________

How would you describe the overall health of your pet?

☐ Excellent ☐ Good ☐ Fair ☐ Poor

How would you describe the activity level of your pet?

☐ Very active ☐ Active ☐ Average activity ☐ Somewhat ☐ Not active

Has your pet been bred? ☐ Yes ☐ No ☐ I am not sure

Is your pet spayed? ☐ Yes ☐ No, the pet is intact ☐ I am not sure

Does your pet have breeding related issues? ☐ Yes ☐ No
